# Exploring the Bidirectional Association Between Migraine and Temporomandibular Disorders: A Systematic Review and Meta‐Analysis

**DOI:** 10.1111/joor.70110

**Published:** 2025-12-24

**Authors:** Marlon Ferreira Dias, Amanda Costa Ferro, Juliana Homem Padilha Spavieri, Túlio Morandin Ferrisse, Daniela Aparecida de Godoi Gonçalves

**Affiliations:** ^1^ Department of Dental Materials and Prosthodontics, School of Dentistry São Paulo State University (UNESP) Araraquara Brazil

**Keywords:** association, meta‐analysis, migraine, migraine disorders, risk factors, systematic review, temporomandibular disorder, temporomandibular joint disorders

## Abstract

**Background:**

Migraine and temporomandibular disorders (TMD) are comorbid conditions that are highly frequent among patients.

**Objective:**

This systematic review with meta‐analysis (MA) aimed to evaluate the bidirectional association between migraine and TMD in adults.

**Methods:**

The protocol was registered with the International Prospective Register of Systematic Reviews. The literature was searched in 5 main databases and 3 grey literature databases from inception until May 12, 2025. The review included observational cross‐sectional studies that based TMD classification on the Research Diagnostic Criteria for TMD (RDC/TMD) or the Diagnostic Criteria for TMD (DC/TMD), and migraine was diagnosed according to any edition of the International Classification of Headache Disorders (ICHD). The risk of bias (Joanna Briggs Institute Meta‐Analysis of Statistics Assessment and Review Instrument tools) and the certainty of evidence (GRADE) were assessed.

**Results:**

From 7329 studies, 17 were included in the qualitative assessment and 6 in the quantitative analysis. Seven studies were classified as low risk of bias, nine as moderate, and one as high. The qualitative assessment demonstrated an association between both conditions. MA revealed that patients with migraine were significantly more likely to have TMD (OR = 6.08; 95% CI: 4.80–7.68), and patients with TMD were more likely to have migraine (OR = 2.64; 95% CI: 2.19–3.18). The certainty of evidence was rated high.

**Conclusion:**

The study identified a strong bidirectional association between migraine and TMD. Clinicians should systematically screen for both conditions, especially in the presence of one of them, to optimize treatment outcomes.

## Graphical abstract

DC/TMD: Diagnostic Criteria for Temporomandibular Disorders; RDC/TMD: Research Diagnostic Criteria for Temporomandibular Disorders; ICHD: International Classification of Headache Disorders. OR: Odds Ratio.

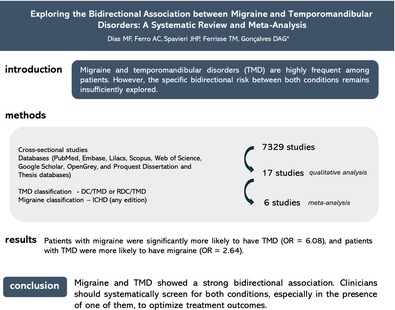

## 1 Introduction

Temporomandibular disorder (TMD) is an umbrella term used to characterise conditions that present pain and dysfunction affecting the masticatory muscles, temporomandibular joint (TMJ), or associated orofacial structures. It is the most common cause of non‐dental pain in the orofacial region [1], with prevalence rates between 10% and 15% [2]. The aetiology is multifactorial and may be caused or influenced by multiple factors, including parafunctional habits, psychosocial alterations, and anatomical and genetic features, among others [3]. It is well known that TMD is frequently associated with various painful and non‐painful comorbid conditions. Among them, primary headaches (PH), especially migraine, are one of the most common comorbid conditions of TMD [4, 5].

Migraine is a prevalent and disabling PH that negatively affects individuals' quality of life [6]. Migraine and TMD share diverse characteristics, such as higher pain sensitization, psychiatric and psychosocial comorbidities, and higher prevalence among women than men [7].

Several cross‐sectional studies [4, 8, 9, 10] have found the relevant association between PH and TMD by suggesting that some of these conditions may share peripheral and central pathophysiological mechanisms [10]. Although previous systematic reviews have explored the association between PH and TMD [11, 12, 13], the specific bidirectional risk between migraine and TMD remains insufficiently explored.

Thus, this systematic review and meta‐analysis (MA) aimed to investigate the potential bidirectional association between migraine and TMD in adults. The null hypothesis is that migraine does not increase the risk of TMD and vice versa.

## 2 Methods

This study was registered with The International Prospective Register of Systematic Reviews (PROSPERO) (registration #CRD42023452872) and reported according to the Preferred Reporting Items for Systematic Reviews and Meta‐analyses (PRISMA).

### 2.1 Eligibility Criteria

The eligibility criteria were defined by the PECOS framework: Population (adults of both sexes), Exposure (the presence of TMD of any type or migraine), Comparison (the absence of the respective condition), Outcome (the prevalence or risk of migraine or TMD), and Study design (observational cross‐sectional studies in any language).

### 2.2 Search Strategy

Three independent reviewers (M.F.D., A.C.F., and J.H.P.) conducted electronic and manual literature searches without date or language restrictions. The search was performed in the following databases: PubMed, Embase, Latin American and Caribbean Health Sciences, Scopus, and Web of Science. The grey literature was searched in Google Scholar, OpenGrey, and Proquest Dissertation and Thesis databases. All searches ended on May 12, 2025 (Table S1; Supporting Information). We also conducted a manual search in the references of the included articles to prevent missing information. All references were managed on appropriate software (Rayyan) and duplicates were removed (EndNote X9 and manual search).

### 2.3 Screening Procedures

The study selection included two phases. In phase 1, the titles and abstracts were read and screened according to the eligibility criteria. In phase 2, the full texts of the studies selected in phase 1 were screened to confirm their eligibility. The second and third authors (A.C.F. and J.H.P.) conducted both phases independently and, at the end of each phase, they verified their agreement regarding the selected articles. The third and fourth authors (M.F.D. and D.A.G.) were contacted to solve any disagreement between the first and second authors.

### 2.4 Data Extraction

The first and second authors (A.C.F. and J.H.P.) collected the main information from the selected studies, and the third author (M.F.D.) verified the data. In case of disagreement, the fourth author (D.A.G.) was consulted to make a final decision.

The information collected from the included studies was general points: author, year of publication, country, study design, sample, type and diagnostic methods of TMD and migraine, and main conclusions. E‐mails were sent to the corresponding authors if the required data were incomplete or unclear in the main text.

### 2.5 Risk of Bias

Two authors (A.C.F. and J.H.P.) independently evaluated the risk of bias among the included studies according to the critical appraisal tools of the Joanna Briggs Institute Meta‐Analysis of Statistics Assessment and Review Instrument [14]. Different questionnaires were selected according to the study design, and they included questions with the following possible answers: ‘Yes,’ ‘No,’ ‘Unclear,’ or ‘Not Applicable.’ The authors categorised the risk of bias as high when the study reached up to 49% of positive answers (Yes), moderate when this rate ranged from 50% to 69%, and low when the study reached more than 70% of ‘Yes’ answers. The authors cross‐checked their answers and, in case of disagreement, the third and fourth authors (M.F.D. and D.A.G.) were consulted.

### 2.6 Statistical Analysis

The quantitative analyses were performed using R software with the ‘Meta’ package, version 3.6.3. The MA evaluated the odds ratio (OR) of TMD in the general population versus in patients with TMD and migraine. An additional meta‐analysis assessed the OR of the general population versus patients with TMD and migraine. The OR was the used measure of effect, and the fixed effect model was applied with a 95% confidence interval (CI).

Heterogeneity was tested using the I2 index, with values > 50% considered substantial or high, and a graphical method proposed by Baujat was also used [15]. In addition, given the data available for the present review, subgroup analyses (similarity among the included studies) and meta‐regression (*n* > 10) could not be performed [16]. Then, the Baujat plot analysis was performed to visualise and quantify the contribution of each individual study to the total heterogeneity. This approach facilitated the identification of potential outliers and sources of heterogeneity [15].

Funnel plots were generated to explore the presence of small‐study effects and potential publication bias across all studies included in the MA. This approach assumes that all included studies estimate a common underlying effect. In this context, smaller studies were expected to show greater variability and therefore to be distributed more widely around the true effect size, whereas larger studies, due to higher precision, were expected to cluster closer to the center of the plot, producing a symmetrical, inverted funnel‐shaped distribution [17].

### 2.7 Quality of Evidence Assessment Using GRADE

The Grade of Recommendation, Assessment, Development, and Evaluation (GRADE) Working Group [18] assessed the quality of evidence for the MA results. This approach evaluates five reasons that may decrease the quality of evidence found in the meta‐analysis (risk of bias, imprecision, inconsistency, indirect evidence, and publication bias) and three factors that may increase the quality of evidence (large effect size, management of confounding factors, dose–response gradient). Each categorization of the quality of evidence for each outcome may be classified as a high, moderate, low, or very low risk of bias.

## 3 Results

### 3.1 Study Selection

**FIGURE 1:**
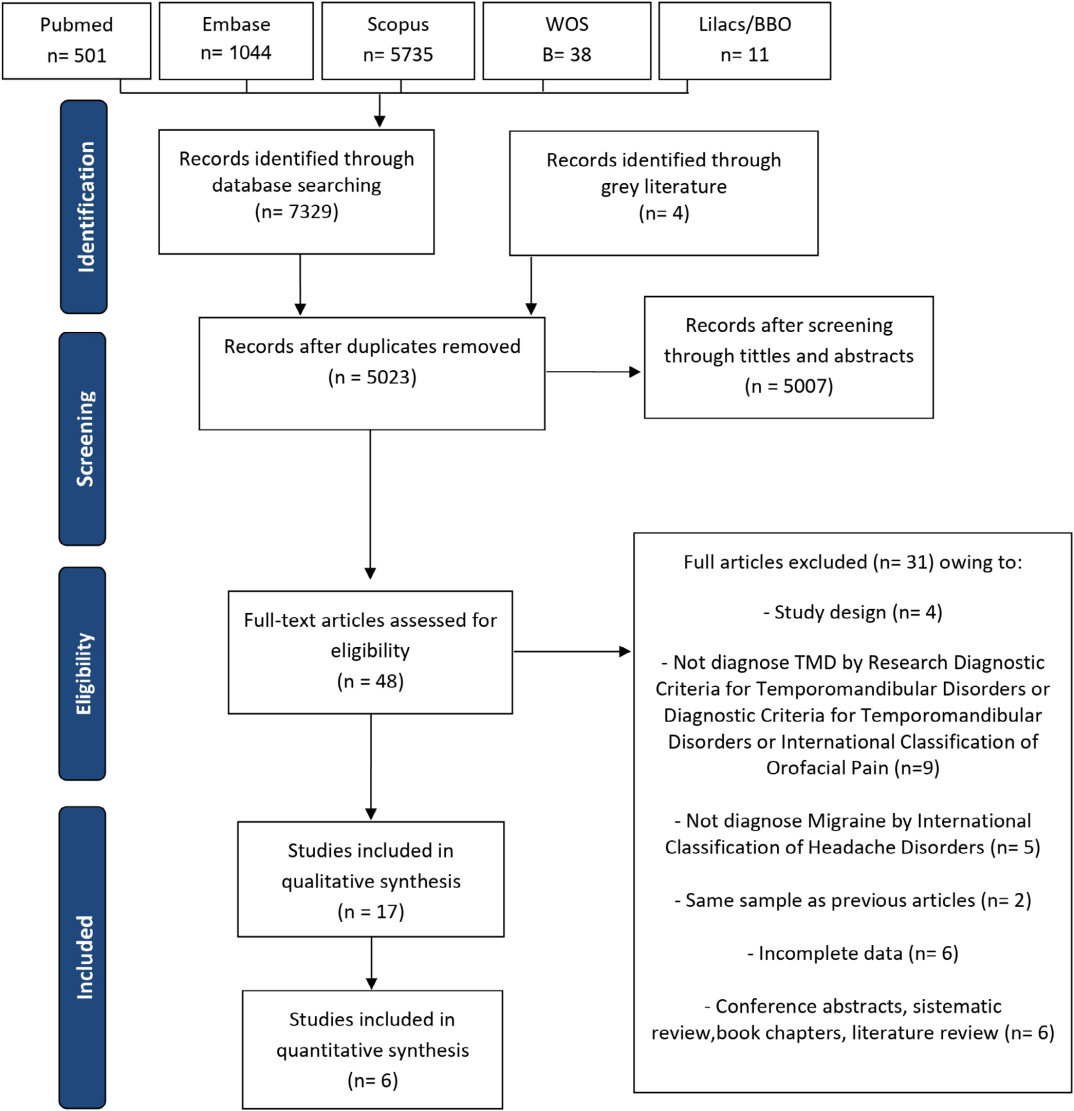
Flowchart of the literature search and selection criteria.

Phase 1 retrieved 7329 citations from electronic databases. After removing duplicates, 5023 titles and abstracts were evaluated according to the eligibility criteria. After excluding 5007 studies, 48 were selected for evaluation in phase 2. Additionally, searches in the grey literature and a manual search in the reference lists of the included articles were also conducted, but no new articles were added. Thirty‐one of the 48 articles previously selected were excluded, resulting in 17 studies for the qualitative analysis. Among these, six were included in the quantitative analysis. Figure 1 presents a flowchart summarising our systematic selection process.

### 3.2 Study Characteristics

The included studies were conducted in nine countries: Brazil, Sweden, USA, Canada, Turkey, Italy, Poland, the Netherlands, and Germany. Sample sizes varied from 45 to 4266 patients. The study designs comprised analytical and descriptive cross‐sectional approaches. Descriptive cross‐sectional studies analysed the data descriptively in a defined population and in a specific period to determine the distribution of one or more variables. Analytical cross‐sectional studies also assessed the relationship between exposure and outcome. We included articles that classified TMD according to the Research Diagnostic Criteria for TMD (RDC/TMD) or the Diagnostic Criteria for TMD (DC/TMD), and migraine according to any edition of the International Classification of Headache Disorders (ICHD). Most studies classified TMD according to the RDC/TMD, and only one applied the DC/TMD criteria. Table 1 provides further details.

**TABLE 1:** Summary of the characteristics of the included articles (*n* = 17).

Author/Country	Sample (Male/Female)	Age	Study design	TMD and migraine diagnosis methods	Sample source	TMD type	Main conclusion
Glaros et al., [30]/USA	5/35	Mean age: 36.1 years	Cross‐sectional	RDC/TMD; ICHD‐2	The staff and faculty of the Kansas City University of Medicine and Biosciences or recruited from the general population of the city of Kansas	Myofascial pain and arthralgia	Headache and TMD patients overlap considerably in diagnosis and oral parafunctional behaviours
Di Paolo et al., [25]/Italy	6/39	Mean age: 34 years	Cross‐sectional	RDC/TMD; ICHD‐2	School of Dentistry and outpatients of the Neurosurgery Institute of Policlinico Umberto I, Sapienza, University of Rome	Muscle disorders, disc displacement, and muscle disorders + disc displacement	The association between migraine and TTH determined a high level of illness disability and may be a risk factor for chronic craniofacial pain and migraine transformation
da Silva Jr. et al., [28]/Brazil	837/768	10 to 93 years	Cross‐sectional	RCD/TMD; ICHD‐2	Individuals from the Family Health Program in a rural area of Brazil interviewed at home by health agents	Myofascial pain and articular dysfunction	Comorbidities among CDH patients were as frequent as reported in convenience samples from tertiary headache centers
Fragoso et al., [29]/Brazil	10/70	Mean age: 47 years	Cross‐sectional prospective	RDC/TMD; ICHD‐2	University‐based headache outpatient clinic		The high prevalence of PH, parafunctional habits, and TMD symptoms, as well as the inadequate use of analgesic drugs, suggest that primary healthcare units need further training in the field of headache and orofacial pain
Troeltzsch et al., [31]/Germany	436/595	Mean age: 49.6 years	Cross‐sectional	RDC/TMD; ICHD‐1	Patients of the oral and maxillofacial surgery practice in Ansbach, Germany	Articular or muscle TMD	Women aged between 30 and 60 years, with muscular pain such as myogenic pain, trigger points, or combinations, tend to present higher headache frequency and intensity
Gonçalves et al., [24]/Brazil	52/248	Mean age: 37.4 years	Analytical cross‐sectional	RDC/TMD; ICHD‐3	UNESP/Araraquara School of Dentistry—Brazil University‐based orofacial pain outpatient clinic	Myofascial, articular, and mixed TMD	TMD, TMD subtypes, and TMD severity were independently associated with specific headache syndromes (migraine and CDH) and frequency, after adjustments
Gonçalves et al., [20]/Brazil	0/91	20 to 55 years	Analytical cross‐sectional	RDC‐TMD; ICHD 2	Hospital and Clinics School of Medicine at Ribeirão Preto, University of São Paulo (HC‐FMRP/USP), University‐based headache outpatient clinic	Muscular TMD, TMJ disc displacement, TMD with arthralgia, osteoarthritis, and arthroses	TMD and migraine were clinically associated. Women with migraine were more likely to have TMD than women without headache, both within muscular and joint diagnoses.
Fernandes et al., [32]/Brazil	45/241	18–76 years	Cross‐sectional	RCD/TMD; ICHD‐2	UNESP/Araraquara School of Dentistry—Brazil University‐based orofacial pain outpatient clinic	Painful and non‐painful TMD	Sleep bruxism and painful TMD considerably increased the risk of episodic migraine, episodic TTH, and especially chronic migraine
Cioffi et al., [26]/Italy	171/610	Mean age (TMD): 35.2 years, (MIG): 36 years	Cross‐sectional	RDC/TMD; ICHD‐edition not informed	Orthodontics and temporomandibular disorders clinic in Naples, Italy	Myofascial pain	Myofascial pain and migraine affect the daily life of adult individuals. The comorbidity of both conditions determined a major impairment
da Silva Jr. et al., [28]/Brazil	38/251	Mean age (TMD): 37.1 years, (MIG): 42.7 years	Cross‐sectional	RCD/TMD; ICHD‐2	Headache Center and Dental Clinic	Myofascial, articular, and mixed TMD	TMD was a relevant comorbidity for migraine, with a difficult clinical distinction from TTH. TTH was more frequent in the Dental Clinic than in the Headache Center
Dahan et al., [9]/Canada	31/149	Mean age: 42.9 years	Cross‐sectional multicenter	RDC/TMD; ID/Migraine	Montreal General Hospital and Massachusetts General Hospital (oral surgery and orofacial pain department)	TMD—myofascial pain or TMD joint/disc disorders	Individuals with m‐TMD had a higher prevalence of self‐reported migraine and CFS, and a higher total number of self‐reported comorbidities than those with n‐TMD
Contreras et al., [27]/Brazil	57/295	18 to 65 years	Cross‐sectional	RDC/TMD; ICHD‐2	UNESP/Araraquara School of Dentistry – Brazil University‐based orofacial pain outpatient clinic	Painful and non‐painful TMD	Individuals with concomitant TMD and migraine had a more severe condition than those with only painful TMD
Wieckiewicz et al., [19]/Poland	64/149	18–84 years	Cross‐sectional	DC/TMD; ICHD‐3	Residents of Polish cities voluntarily answered questions in shopping malls	Myofascial and articular TMD	Headache was more frequent among participants with a painful TMD. The relationship between TMD and headaches was not significant
Sharma et al., [8]/Sweden	2373/1893	18 to 44 years	Cross‐sectional	DC/TMD; ICHD 3	University of Buffalo, University of Florida, University of Maryland, and University of North Carolina	TMD myalgia or arthralgia	The overlap between COPC and characteristics typically regarded as painful TMD had implications for treatment targeted at the local TMD condition and the broader pain disorder underlying the COPC(s)
Memmedova et al., [23]/Turkey	90/258	18 to 66 years	Cross‐sectional outpatient‐based	RDC/TMD; ICHD‐3	Neurology Clinic of the Istanbul Training and Research Hospital	Not informed	The rate of TMD among primary headache patients was as high as 25%, with a statistically significant association. TMD was more frequent among migraine patients than among those with TTH
Proença et al., [22]/Brazil	24/105	Mean age: 36.6 years	Cross‐sectional	RDC/TMD; ICHD‐3	Araraquara School of Dentistry—Brazil University‐based orofacial pain outpatient clinic	Painful and non‐painful TMD	Individuals with painful TMD and comorbidities presented more clinical features of CPP than those affected only by TMD
Calixtre et al., [21]/Brazil	0/95	Mean age: 31.7 years	Observational cross‐sectional	DC/TMD; ICHD‐3	Patients from social media	Painful and non‐painful TMD	The positive association between TMD and the CSI score was confounded by migraine, depression symptoms, widespread pain, and parafunctional oral habits

Abbreviations: CDH, chronic daily headache; CFS, chronic fatigue syndrome; COPC, chronic overlapping pain conditions; CPP, chronic primary pain; CSI, central sensitization inventory; DC/TMD, diagnostic criteria for temporomandibular disorders; ICHD, international classification of headache disorders; PH, primary headache; RDC/TMD, research diagnostic criteria for temporomandibular disorders; TTH, tension‐type headache.

### 3.3 Risk of Bias Within Studies

The study applied two tools of the Joanna Briggs Institute Meta‐Analysis of Statistics Assessment and Review Instrument to evaluate the risk of bias according to analytical and descriptive cross‐sectional studies [14].

Fourteen articles were classified as analytical cross‐sectional, six of which had a low risk of bias [8, 9, 19, 20, 21, 22], and eight had a moderate risk of bias [23, 24, 25, 26, 27, 28, 29, 30]. Among the three descriptive cross‐sectional articles [31, 32, 33], one was classified as a low risk of bias [32], one as moderate [31], and one as a high [33] risk of bias. Tables 2 and 3 present detailed information on the risk of bias assessment.

**TABLE 2:** Risk of bias assessed by the Joanna Briggs Institute critical appraisal tool: analytical cross‐sectional studies.

Question	Glaros et al. [30]	Di Paolo et al. [25]	da Silva Jr. et al. [28]	Fragoso et al. [29]	Gonçalves et al. [24]	Gonçalves et al. [20]	Cioffi et al. [26]	Dahan et al. [9]	Contreras et al. [27]	Wieckiewicz et al. [19]	Sharma et al. [8]	Memmedova et al. [23]	Proença et al. [22]	Calixtre et al. [21]
Were the criteria for inclusion in the sample clearly defined?	Unclear	Yes	Unclear	Unclear	Unclear	Yes	Unclear	Yes	Yes	Yes	Yes	Unclear	Yes	Yes
Were the study subjects and the setting described in detail?	Yes	Unclear	Yes	Yes	Yes	Yes	Unclear	Yes	Unclear	Yes	Yes	Unclear	Yes	Yes
Was the exposure measured in a valid and reliable way?	Yes	Yes	Yes	Yes	Yes	Yes	Yes	Yes	Yes	Yes	Yes	Yes	Yes	Yes
Were objective, standard criteria used for measurement of the condition?	Yes	Yes	Yes	Yes	Yes	Yes	Yes	Yes	Yes	Yes	Yes	Yes	Yes	Yes
Were Confounding Factors Identified?	No	No	No	No	No	No	No	No	No	No	No	No	No	Yes
Were strategies to deal with confounding factors stated?	No	No	No	No	No	No	No	No	No	No	No	No	No	No
Were the outcomes measured in a valid and reliable way?	Yes	Yes	Yes	Yes	Yes	Yes	Yes	Yes	Yes	Yes	Yes	Yes	Yes	Yes
Was appropriate statistical analysis used?	Yes	Yes	Yes	Yes	Yes	Yes	Yes	Yes	Yes	Yes	Yes	Yes	Yes	Yes
% Yes/Risk	62.5/Moderate	62.5/Moderate	62.5/Moderate	62.5/Moderate	62.5/Moderate	75/Low	50/Moderate	75/Low	62.5/Moderate	75/Low	75/Low	50/Moderate	75/Low	87.5/Low

**TABLE 3:** Risk of bias assessed by the Joanna Briggs Institute critical appraisal tool: descriptive cross‐sectional studies.

Question	Troeltzsch et al. [31]	Fernandes et al. [32]	da Silva Júnior et al. [28]
Was the study based on a random or pseudo‐random sample?	Yes	No	No
Were the criteria for inclusion in the sample clearly defined?	Yes	Yes	No
Were confounding factors identified and strategies to deal with them stated?	No	Yes	No
Were outcomes assessed using objective criteria?	Yes	Yes	Yes
If comparisons are being, was there a sufficient description of the groups?	No	Yes	No
Was follow‐up carried out over a sufficient period?	NA	NA	NA
Were the outcomes of people who withdrew described and included in the analysis?	Yes	Yes	Yes
Were outcomes measured in a reliable way?	Yes	Yes	Yes
Was appropriate statistical analysis used?	Yes	Yes	Yes
% Yes/Risk	66.6/Moderate	77.7/Low	44.4/High

Abbreviation: NA, not applicable.

### 3.4 Association Between TMD and Migraine

Several studies assessed the prevalence of PH in individuals with TMD [9, 27, 30, 31]. Moreover, sex‐related differences were identified, as women without headache exhibited a substantially lower TMD prevalence (33.3%) than those diagnosed with migraine (86.8%) and chronic migraine (91.3%) [20].

Compared with other PH, migraine exhibits a stronger association with TMD [19, 23, 28, 32, 34]. This association varies depending on the TMD subtypes [33] and the level of chronicity (Graded Chronic Pain Scale—GCPS). In a previous study, individuals with severe myofascial TMD were significantly more likely to have migraine than those with other TMD types or lower GCPS scores [24]. Furthermore, the frequency of headache was nearly twice as high in individuals with TMD than in those without TMD: 48.3% vs. 25.3%, respectively [19].

Migraine‐related disability may also be higher in patients presenting the two conditions concomitantly than in those with isolated migraine [25]. Comorbidity is also related to a higher social impairment in daily life [26]. A previous study also demonstrated that the risk of PH occurrence in individuals with painful TMD significantly increased the risk of chronic migraine (OR = 30.1; 95% CI: 3.58–252.81), followed by episodic migraine (OR = 3.7; 95% CI: 1.46–9.16) [32]. Moreover, somatic symptoms and bruxism may be relevant confounders of the association between TMD and migraine [34]. Additionally, migraine was addressed as a confounding factor in the association between TMD and central sensitization [21].

### 3.5 Meta‐Analysis Results and Quality of Evidence

**FIGURE 2:**
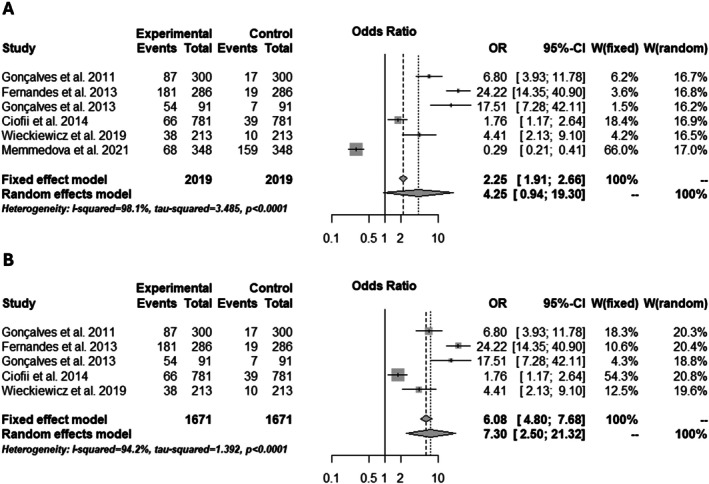
Risk of TMD in patients with migraine. (A) Overall risk; (B) Risk after the subgroup analysis.

The MA highlighted that patients with migraine are 2.25 [1.91; 2.66] times more likely to develop TMD (Figure 2); however, the funnel plot showed publication biases. The Baujat test was applied to explore data heterogeneity and potential publication bias, helping identify the source of heterogeneity. Subsequently, a subgroup meta‐analysis was performed, excluding the article responsible for the overall heterogeneity. The new findings indicated that patients with migraine are 6.08 [4.80; 7.68] times more likely to develop TMD (Figure 2). However, a publication bias and a high level of heterogeneity (*I* ^2^ = 94.2%) remain.

**FIGURE 3:**
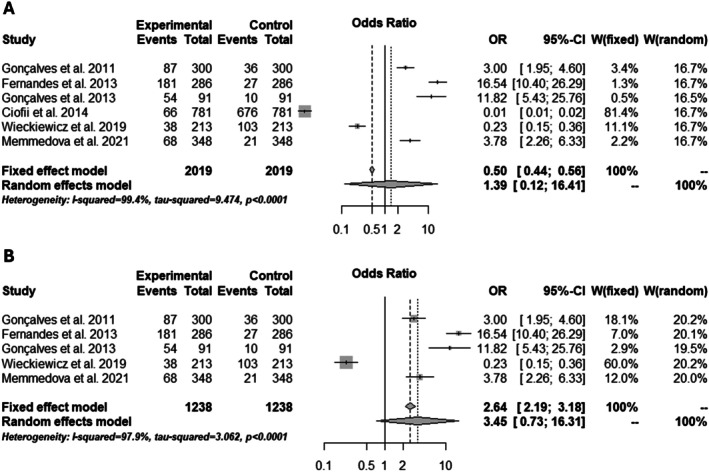
Risk of migraine in patients with TMD. (A) Overall risk; (B) Risk after the subgroup analysis.

Another meta‐analysis investigated the risk of migraine among patients with TMD. Initially, the analysis showed an unexpected protective association [0.50 (0.44; 0.56)] (Figure 3), with the funnel plot indicating publication biases. The Baujat test was also conducted and, after removing the source of heterogeneity, this association was reversed, indicating that patients with TMD are more likely to present an associated migraine [2.64 (2.19; 3.18)] (Figure 3).

The absence of sample size calculation in almost all articles included in the MA downgraded the quality of evidence. However, due to the large effect size (OR > 2), the meta‐analysis result was considered at a high level of certainty [18].

## 4 Discussion

This systematic review investigated the potential bidirectional association between migraine and temporomandibular disorder (TMD) in cross‐sectional studies. The meta‐analysis demonstrated a positive and bidirectional association between migraine and TMD. The qualitative assessment also identified a substantial number of studies supporting a strong association between these conditions. Therefore, the null hypothesis of this study was rejected.

Migraine is a common disabling primary headache (PH) disorder defined as a headache not caused by an underlying disease or condition [8]. It is frequently associated with TMD, and the comorbidity may involve shared underlying mechanisms [35, 36]. Previous systematic reviews have associated PH with TMD [11, 12, 13].

A previous systematic review [11] found a significant association between TMD and PH, except in cases of non‐painful articular TMD. Another study reported that only mixed TMD demonstrated a moderate association with PH, indicating no significant correlation for isolated muscle or articular TMD [12]. The meta‐analysis in our study presented a strong association between TMD and migraine; however, we did not analyse the correlation between migraine and TMD subtypes separately. It is also noteworthy that most articles included in the meta‐analysis involved painful TMD, highlighting the relevance of painful muscle TMD in this comorbidity. The qualitative analysis and the meta‐analysis demonstrated a strong association between these conditions, although most included studies were classified as a low or moderate risk of bias.

While the association between TMD and migraine has not been fully explained, some hypotheses involving different factors have been formulated. A relevant one regards the involvement of the shared pain pathway and mechanisms. The TMD‐related mechanisms include peripheral sensitization in musculoskeletal structures, which may reduce the nociceptive threshold and sustain local pain [37]. This context may interfere by aggravating, perpetuating, or even triggering migraine [38]. Additionally, the antidromic release of neurotransmitters, such as substance P and calcitonin gene‐related peptide (CGRP), is crucial to the pathophysiology of both disorders and may help establish the central sensitization process [39]. However, this study did not assess the correlation of biomarkers to TMD and migraine.

This systematic review has limitations and strengths. One limitation concerns the confounding factors, which are recurrent among observational studies. Only two studies [21, 32] in our review reported confounders, potentially introducing biases into the results. Additionally, the meta‐analysis included only six studies, involving a total population of 2019 individuals. The small number of articles with available cross‐sectional data in the MA may compromise the results regarding the relationship between both disorders. One of the strengths of the present study is the inclusion of a relatively high number of full texts in the qualitative assessment (*n* = 17), comprising 10 131 individuals. The included studies also showed a low risk of bias and high methodological quality, indicating a strong quality of evidence in our systematic review. Furthermore, analysing the bidirectional relationship between both conditions represents another strength, as previous systematic reviews investigated the risk of TMD in patients with PH or vice versa, but not both ways [11, 40].

Therefore, our findings support the recommendation that healthcare professionals working with orofacial pain and headache should be prepared to identify migraine and TMD symptoms, especially in the presence of one of them. The existence of one condition may increase the likelihood of the other, and the best practice in such cases is to therapeutically address both cases simultaneously. This approach may lead to a better prognosis and significantly improve both conditions [41], ultimately enhancing the patients' quality of life. Furthermore, additional studies are required to elucidate the various factors related to this comorbidity by addressing the underlying mechanisms and conducting longitudinal investigations that explain the progression and interactions of both conditions.

## 5 Conclusion

The qualitative analysis demonstrated a strong association between TMD and migraine, while the meta‐analysis revealed a positive bidirectional association between the two conditions.

## Author Contributions

**Marlon Ferreira Dias:** conceptualization, data curation, methodology, formal analysis, investigation, and writing – original draft preparation. **Amanda Costa Ferro:** formal analysis, investigation, and methodology. **Juliana Homem Padilha Spavieri:** formal analysis, investigation, and methodology. **Túlio Morandin Ferrisse:** formal analysis and methodology. **Daniela Aparecida de Godoi Gonçalves:** conceptualization, methodology, formal analysis, project administration, supervision, and writing – review and editing.

## Conflicts of Interest

The authors declare no conflicts of interest.

## Supporting information


**Table S1:** Database search strategy.

## Data Availability

Data will be provided upon request.
