# Supplementary material for: Exploring the Bidirectional Association Between Migraine and Temporomandibular Disorders: A Systematic Review and Meta‐Analysis
Source: J Oral Rehabil. 2025 Dec 24;53(3):794–806. doi: 10.1111/joor.70110 (PMC12902198; doi:10.1111/joor.70110)
Supplement: Supplementary file 1 — Table S1: Database search strategy. [file JOOR-53-794-s001.docx]

**Table S1**. Database search strategy.

| **Database** | **Search** |
| --- | --- |
| **PubMed** | (“Disorder, Migraine” OR “Disorders, Migraine” OR “Migraine Disorder” OR Migraine OR Migraines OR “Migraine Headache” OR “Headache, Migraine” OR “Headaches, Migraine” OR “Migraine Headaches” OR “headache, migrainous” OR “migraine disorders” OR “migrainous headache”) AND (“TMJ syndrome” OR “temporomandibular joint syndrome” OR TMD OR “costen's syndrome” OR “costen syndrome” OR “craniomandibular pain” OR “craniomandibular disorder” OR “craniomandibular dysfunction” OR “orofacial pain” OR “temporomandibular disorder” OR “temporomandibular disorders” OR “temporomandibular pain” OR “Disorder, Temporomandibular Joint” OR “Disorders, Temporomandibular Joint” OR “Joint Disorder, Temporomandibular” OR “Joint Disorders, Temporomandibular” OR “Temporomandibular Joint Disorder” OR “TMJ Disorders” OR “Disorder, TMJ” OR “Disorders, TMJ” OR “TMJ Disorder” OR “Temporomandibular Disorders” OR “Disorder, Temporomandibular” OR “Disorders, Temporomandibular” OR “Temporomandibular Disorder” OR “Temporomandibular Joint Diseases” OR “Disease, Temporomandibular Joint” OR “Diseases, Temporomandibular Joint” OR “Joint Disease, Temporomandibular” OR “Joint Diseases, Temporomandibular” OR “Temporomandibular Joint Disease” OR “TMJ Diseases” OR “Disease, TMJ” OR “Diseases, TMJ” OR “TMJ Disease”) |
| **Embase** | (“Disorder, Migraine” OR “Disorders, Migraine” OR “Migraine Disorder” OR Migraine OR Migraines OR “Migraine Headache” OR “Headache, Migraine” OR “Headaches, Migraine” OR “Migraine Headaches” OR “headache, migrainous” OR “migraine disorders” OR “migrainous headache”) AND (“TMJ syndrome” OR “temporomandibular joint syndrome” OR TMD OR “costen's syndrome” OR “costen syndrome” OR “craniomandibular pain” OR “craniomandibular disorder” OR “craniomandibular dysfunction” OR “orofacial pain” OR “temporomandibular disorder” OR “temporomandibular disorders” OR “temporomandibular pain” OR “Disorder, Temporomandibular Joint” OR “Disorders, Temporomandibular Joint” OR “Joint Disorder, Temporomandibular” OR “Joint Disorders, Temporomandibular” OR “Temporomandibular Joint Disorder” OR “TMJ Disorders” OR “Disorder, TMJ” OR “Disorders, TMJ” OR “TMJ Disorder” OR “Temporomandibular Disorders” OR “Disorder, Temporomandibular” OR “Disorders, Temporomandibular” OR “Temporomandibular Disorder” OR “Temporomandibular Joint Diseases” OR “Disease, Temporomandibular Joint” OR “Diseases, Temporomandibular Joint” OR “Joint Disease, Temporomandibular” OR “Joint Diseases, Temporomandibular” OR “Temporomandibular Joint Disease” OR “TMJ Diseases” OR “Disease, TMJ” OR “Diseases, TMJ” OR “TMJ Disease”) |
| **Latin American and Caribbean Health Sciences** | (“Transtornos de Enxaqueca” OR “Migraine Disorders” OR “Trastornos Migrañosos” OR “Cefaleia Enxaquecosa” OR “Estado de Mal Enxaquecoso” OR Migrânea OR “Síndromes Enxaquecosas” OR “Síndromes de Enxaqueca” OR “Transtornos da Enxaqueca” OR Enxaqueca) AND (“Síndrome da Disfunção da Articulação Temporomandibular” OR “Temporomandibular Joint Dysfunction Syndrome” OR “Síndrome de la Disfunción de Articulación Temporomandibular” OR “Síndrome Miofascial de Disfunção Dolorosa Temporomandibular” OR “Síndrome da ATM” OR “Síndrome da Articulação Temporomandibular” OR DTM OR “Disfunção da Articulação Temporomandibular” OR ATM OR “Desordens da ATM”) |
| **Scopus** | (“Disorder, Migraine” OR “Disorders, Migraine” OR “Migraine Disorder” OR Migraine OR Migraines OR “Migraine Headache” OR “Headache, Migraine” OR “Headaches, Migraine” OR “Migraine Headaches” OR “headache, migrainous” OR “migraine disorders” OR “migrainous headache”) AND (“TMJ syndrome” OR “temporomandibular joint syndrome” OR TMD OR “costen's syndrome” OR “costen syndrome” OR “craniomandibular pain” OR “craniomandibular disorder” OR “craniomandibular dysfunction” OR “orofacial pain” OR “temporomandibular disorder” OR “temporomandibular disorders” OR “temporomandibular pain” OR “Disorder, Temporomandibular Joint” OR “Disorders, Temporomandibular Joint” OR “Joint Disorder, Temporomandibular” OR “Joint Disorders, Temporomandibular” OR “Temporomandibular Joint Disorder” OR “TMJ Disorders” OR “Disorder, TMJ” OR “Disorders, TMJ” OR “TMJ Disorder” OR “Temporomandibular Disorders” OR “Disorder, Temporomandibular” OR “Disorders, Temporomandibular” OR “Temporomandibular Disorder” OR “Temporomandibular Joint Diseases” OR “Disease, Temporomandibular Joint” OR “Diseases, Temporomandibular Joint” OR “Joint Disease, Temporomandibular” OR “Joint Diseases, Temporomandibular” OR “Temporomandibular Joint Disease” OR “TMJ Diseases” OR “Disease, TMJ” OR “Diseases, TMJ” OR “TMJ Disease”) |
| **Web of Science** | (“Disorder, Migraine” OR “Disorders, Migraine” OR “Migraine Disorder” OR Migraine OR Migraines OR “Migraine Headache” OR “Headache, Migraine” OR “Headaches, Migraine” OR “Migraine Headaches” OR “headache, migrainous” OR “migraine disorders” OR “migrainous headache”) AND (“TMJ syndrome” OR “temporomandibular joint syndrome” OR TMD OR “costen's syndrome” OR “costen syndrome” OR “craniomandibular pain” OR “craniomandibular disorder” OR “craniomandibular dysfunction” OR “orofacial pain” OR “temporomandibular disorder” OR “temporomandibular disorders” OR “temporomandibular pain” OR “Disorder, Temporomandibular Joint” OR “Disorders, Temporomandibular Joint” OR “Joint Disorder, Temporomandibular” OR “Joint Disorders, Temporomandibular” OR “Temporomandibular Joint Disorder” OR “TMJ Disorders” OR “Disorder, TMJ” OR “Disorders, TMJ” OR “TMJ Disorder” OR “Temporomandibular Disorders” OR “Disorder, Temporomandibular” OR “Disorders, Temporomandibular” OR “Temporomandibular Disorder” OR “Temporomandibular Joint Diseases” OR “Disease, Temporomandibular Joint” OR “Diseases, Temporomandibular Joint” OR “Joint Disease, Temporomandibular” OR “Joint Diseases, Temporomandibular” OR “Temporomandibular Joint Disease” OR “TMJ Diseases” OR “Disease, TMJ” OR “Diseases, TMJ” OR “TMJ Disease”) |
| **Google Scholar** | (“Transtornos de Enxaqueca” OR “Migraine Disorders” OR “Trastornos Migrañosos” OR “Cefaleia Enxaquecosa” OR “Estado de Mal Enxaquecoso” OR Migrânea OR “Síndromes Enxaquecosas” OR “Síndromes de Enxaqueca” OR “Transtornos da Enxaqueca” OR Enxaqueca) AND (“Síndrome da Disfunção da Articulação Temporomandibular” OR “Temporomandibular Joint Dysfunction Syndrome” OR “Síndrome de la Disfunción de Articulación Temporomandibular” OR “Síndrome Miofascial de Disfunção Dolorosa Temporomandibular” OR “Síndrome da ATM” OR “Síndrome da Articulação Temporomandibular” OR DTM OR “Disfunção da Articulação Temporomandibular” OR ATM OR “Desordens da ATM”) |
| **OpenGrey** | (headache OR migraine) AND ("temporomandibular disorder" OR TMD) |
| **Proquest Dissertation and Thesis** | (headache OR migraine) AND ("temporomandibular disorder" OR TMD) |
